# Supplementary material for: Death Induced by Survival gene Elimination (DISE) correlates with neurotoxicity in Alzheimer’s disease and aging
Source: Nat Commun. 2024 Jan 18;15:264. doi: 10.1038/s41467-023-44465-8 (PMC10796375; doi:10.1038/s41467-023-44465-8)
Supplement: Supplementary file 3 — Reporting Summary [file 41467_2023_44465_MOESM3_ESM.pdf]

nature portfolio

Corresponding author(s): Marcus Peter

Last updated by author(s): Dec 8, 2023

Reporting Summary

Nature Portfolio wishes to improve the reproducibility of the work that we publish. This form provides structure for consistency and transparency in reporting. For further information on Nature Portfolio policies, see our Editorial Policies and the Editorial Policy Checklist.

Statistics

For all statistical analyses, confirm that the following items are present in the figure legend, table legend, main text, or Methods section.

n/a	Confirmed	
☐	☒	The exact sample size (*n*) for each experimental group/condition, given as a discrete number and unit of measurement
☐	☒	A statement on whether measurements were taken from distinct samples or whether the same sample was measured repeatedly
☐	☒	The statistical test(s) used AND whether they are one- or two-sided<br>*Only common tests should be described solely by name; describe more complex techniques in the Methods section.*
☒	☐	A description of all covariates tested
☒	☐	A description of any assumptions or corrections, such as tests of normality and adjustment for multiple comparisons
☐	☒	A full description of the statistical parameters including central tendency (e.g. means) or other basic estimates (e.g. regression coefficient) AND variation (e.g. standard deviation) or associated estimates of uncertainty (e.g. confidence intervals)
☐	☒	For null hypothesis testing, the test statistic (e.g. *F*, *t*, *r*) with confidence intervals, effect sizes, degrees of freedom and *P* value noted<br>*Give P values as exact values whenever suitable.*
☒	☐	For Bayesian analysis, information on the choice of priors and Markov chain Monte Carlo settings
☒	☐	For hierarchical and complex designs, identification of the appropriate level for tests and full reporting of outcomes
☐	☒	Estimates of effect sizes (e.g. Cohen's *d*, Pearson's *r*), indicating how they were calculated

Our web collection on statistics for biologists contains articles on many of the points above.

Software and code

Policy information about availability of computer code

Data collection	To collect IncuCyte data IncuCyte Zoom software (Version 2016A.lnk) was used.
Data analysis	Microsoft Excel v.16; SPOROS (PLOS Comp Biol. 18, e1010022); StatPlus v.8.0.4; Stata v.14, Nikon Advanced Research (NIS) Elements software

For manuscripts utilizing custom algorithms or software that are central to the research but not yet described in published literature, software must be made available to editors and reviewers. We strongly encourage code deposition in a community repository (e.g. GitHub). See the Nature Portfolio guidelines for submitting code & software for further information.

Data

Policy information about availability of data

All manuscripts must include a data availability statement. This statement should provide the following information, where applicable:

- Accession codes, unique identifiers, or web links for publicly available datasets
- A description of any restrictions on data availability
- For clinical datasets or third party data, please ensure that the statement adheres to our policy

RNA seq data were deposited at GEO under accession number GSE213138. All source data are included in separate file. All other data generated and analyzed during this study are included in this published article and its supplementary information files, or are available upon request.

Research involving human participants, their data, or biological material

Policy information about studies with human participants or human data. See also policy information about sex, gender (identity/presentation), and sexual orientation and race, ethnicity and racism.

Reporting on sex and gender	Gender for all human cell lines (when available) , mutant mice and human specimens are reported.
Reporting on race, ethnicity, or other socially relevant groupings	NA
Population characteristics	Three cognitive normal participants (female age 83, male age 87, female age 100) and three SuperAgers (male age 90, female age 85, female age 82).
Recruitment	NA
Ethics oversight	Study was approved by the Northwestern University Institutional Review Board and in accordance with the Helsinki Declaration

Note that full information on the approval of the study protocol must also be provided in the manuscript.

Field-specific reporting

Please select the one below that is the best fit for your research. If you are not sure, read the appropriate sections before making your selection.

- [x] Life sciences
- [ ] Behavioural & social sciences
- [ ] Ecological, evolutionary & environmental sciences

For a reference copy of the document with all sections, see nature.com/documents/nr-reporting-summary-flat.pdf

Life sciences study design

All studies must disclose on these points even when the disclosure is negative.

Sample size	Sample sizes (between 3 and 6) were used in most assays as 3 samples are usually used in these types of assays. Some of the RNA seq data data points were done in duplicate. This was done for tissue availability and cost reasons. In all other RNA Seq analyses sample size was 3 or 4. The only sample with n=1 per condition was the aged IPSC derived neurons as these cells are very difficult to maintain at the large numbers required for this type of analysis.
Data exclusions	Data excluded in IncuCyte experiments or ATP measurements when wells were unreadable.
Replication	The number of technical and biological repeats are given in the figure legends.
Randomization	All read outs were unbiased due to the types of analyses. Randomization was therefore not needed.
Blinding	As no subjective analyses are included in the study, blinding of investigators was not required. All samples were analyzed by instruments not humans. Sample preparation was also done in an unbiased way.

Reporting for specific materials, systems and methods

We require information from authors about some types of materials, experimental systems and methods used in many studies. Here, indicate whether each material, system or method listed is relevant to your study. If you are not sure if a list item applies to your research, read the appropriate section before selecting a response.

Materials & experimental systems

n/a	Involved in the study	
	x	Antibodies
	x	Eukaryotic cell lines
x		Palaeontology and archaeology
	x	Animals and other organisms
	x	Clinical data
x		Dual use research of concern
x		Plants

Methods

n/a	Involved in the study	
x		ChIP-seq
x		Flow cytometry
x		MRI-based neuroimaging

Antibodies

Antibodies used	Argonaute-2 rabbit pAb (Abcam #ab32381, 1:1000), tyrosine hydroxylase r abbit pAb (TH)(Cell Signaling #2792, 1:1000), GAP43

	(D9C8) rabbit mAb (Cell Signaling #8945, 1:1000), tau1 mouse mAb, clone PC1C6 (Millipore Sigma #MAB3420, 5 µg/mL), drosha (D28B1) rabbit mAb (Cell Signaling #3364, 1:1000), dicer (D38E7) rabbit mAb (Cell Signaling #5362, 1:1000), goat anti-rabbit secondary Ab (Southern Biotech #4030-05, 1:10,000), goat anti-mouse 2°Ab (Southern Biotech #1070-05, 1:10,000), HRP-conjugated β-actin (C4) mouse mAb (Santa Cruz Biotechnology #Sc-47778, 1:10,000), phospho-histone H2A.X (Ser139) (20E3) rabbit mAb (Cell Signaling #9718, 1:1000), neuronal class III tubulin (TUJ1, Millipore #T8660, 1:1000) with secondary Ab alexafluor goat anti-mouse IgG2b 488 (Invitrogen #A-21141, 1:1000), glutamatergic neuronal marker vesicular glutamate transporter (Vglut1, Synaptic Systems #135 304, 1:500) with secondary Ab alexafluor 555 goat anti-guinea pig Invitrogen #A-21435, 1:1000), LMX1A (rabbit pAb, Millipore #AB10533, 1:1000 IF), tyrosine hydroxylase (pAb, Millipore #657012, 1:1000 IF), HNF-3 RY7 (FOXA2) (mouse mAb, Santa Cruz # sc-101060, 1:100 IF), TUJ1 (mouse mAb, Covance #MMS-435P, 1:2000) used for WB or IF (used in Supplementary Fig. 4) with secondary Abs anti-mouse or rabbit IgG conjugated Alexa 488 (1:400) or Alexa 568 (1:200) (Life Technologies).
Validation	All 14 antibodies used are either widely used and validated or validated in this study by using knock out cell lines. In addition, we have validated antibodies by matching their expected subcellular location with what we observe (nuclear vs cytoplasmic vs axonal), and testing their reactivity in cells that do not express these proteins. For example, the antibodies to characterize iPSC derived neurons detect proteins that are absent in stem cells but present in midbrain neurons. Therefore, we have validated that they do not react with stem cells.

Eukaryotic cell lines

Policy information about cell lines and Sex and Gender in Research

Cell line source(s)	Human neuroblastoma cell lines; SH-SY5Y (# CRL-2266TM) (SH cells) were purchased from ATCC and NB7 were kindly provided by Dr. Jill Lahti (St. Jude Children's Research Hospital, Memphis, TN).
Authentication	NB7 cells are validated by the absence of caspase-8. SH-SY5Y cells were purchased from the ATCC. The latter cell line was also authenticated by the ability to differentiate into neuron like cells with upregulation of neuronal markers and tau. In addition, both cell lines were authenticated by STR profiling.
Mycoplasma contamination	All cell lines tested were negative for mycoplasma contamination.
Commonly misidentified lines (See ICLAC register)	Used cell lines are not part of this data set.

Animals and other research organisms

Policy information about studies involving animals; ARRIVE guidelines recommended for reporting animal research, and Sex and Gender in Research

Laboratory animals	Young and old mice (2 months and 18 months) were in the C57BL/6J background. Rubicon k.o. (and heterozygous control mice) were in a mixed C57BL/6J/SJL background. Their age was 6 or 8 months at analysis. Tau tg mice were in the C57BL/6J background (backcrossed for at least five generations) and there age was 3 months at analysis. To ensure optimal living conditions and the well-being of the animals, mice were housed in Individually Ventilated Cages (IVCs) equipped with additional environmental enrichment devices, such as mouse lofts and mezzanines. The housing conditions included a 12-hour light-dark cycle, with careful maintenance of relative humidity within the range of 40 to 60%. The temperature settings for the mouse rooms were controlled and set at 65-75°F (~18-23°C).
Wild animals	No wild animals were used in the study.
Reporting on sex	Gender is given in the manuscript.
Field-collected samples	No field collected samples were used in the study.
Ethics oversight	All animal experiments were approved and conducted in accordance with IACUC regulations at Northwestern University, St. Jude's Children's Hospital and University of South Florida, Tampa.

Note that full information on the approval of the study protocol must also be provided in the manuscript.

Clinical data

Policy information about clinical studies
All manuscripts should comply with the ICMJEguidelines for publication of clinical research and a completedCONSORT checklist must be included with all submissions.

Clinical trial registration	NA
Study protocol	NA
Data collection	NA
Outcomes	NA
